# Treating heart failure by targeting the vagus nerve

**DOI:** 10.1007/s10741-024-10430-w

**Published:** 2024-08-09

**Authors:** Francesco Gentile, Giulia Orlando, Sabrina Montuoro, Yu Fu Ferrari Chen, Vaughan Macefield, Claudio Passino, Alberto Giannoni, Michele Emdin

**Affiliations:** 1 Health Science Interdisciplinary Center, Scuola Superiore Sant’Anna, Piazza Martiri Della Libertà 33, 56127 Pisa, Italy; 2 Cardiology and Cardiovascular Medicine Division, Fondazione Monasterio, Via G. Moruzzi 1, 56124 Pisa, Italy; 3 Neuroscience Department, Monash University, Melbourne, Australia

**Keywords:** Parasympathetic nervous system, Vagus nerve, Vagus nerve stimulation, Neuromodulation, Heart rate variability, Baroreflex sensitivity, Heart failure

## Abstract

Increased sympathetic and reduced parasympathetic nerve activity is associated with disease progression and poor outcomes in patients with chronic heart failure. The demonstration that markers of autonomic imbalance and vagal dysfunction, such as reduced heart rate variability and baroreflex sensitivity, hold prognostic value in patients with chronic heart failure despite modern therapies encourages the research for neuromodulation strategies targeting the vagus nerve. However, the approaches tested so far have yielded inconclusive results. This review aims to summarize the current knowledge about the role of the parasympathetic nervous system in chronic heart failure, describing the pathophysiological background, the methods of assessment, and the rationale, limits, and future perspectives of parasympathetic stimulation either by drugs or bioelectronic devices.

## Introduction

The autonomic nervous system (ANS) plays a key role in the neuroendocrine control of the body by adapting vegetative functions to support homeostasis.

In healthy conditions, the sympathetic (SNS) and parasympathetic nervous systems (PSNS) participate in cardiovascular control in a complementary and, at least partially, opposite fashion. Of note, while the heart receives both innervations, the resistance vessels of the systemic circulation are exclusively controlled by the SNS.

On the other hand, autonomic imbalance is a determinant of cardiovascular disorders, such as chronic heart failure (CHF). In the acute setting, chemoreflex activation and baroreflex deactivation induce PSNS withdrawal and SNS overactivation as compensatory mechanisms to maintain perfusion and respiratory efficiency. Over the long term, SNS predominance is maladaptive and, also by activating the renin–angiotensin–aldosterone system (RAAS), favors salt and water retention, cardiac remodeling, and life-threatening arrhythmias [1].

Accordingly, several markers of autonomic dysfunction retain prognostic significance [2], while counteracting SNS improves survival in patients with CHF and reduced left ventricular ejection fraction (LVEF) [3]. On the other hand, because of the contradictory findings from clinical trials, the role of neurohormonal systems seems less consistent in patients with CHF and preserved LVEF, as reviewed in [4]. Indeed, also the usefulness of beta-blockers has been questioned in these patients, since they may contribute to chronotropic incompetence and, consequently, to exercise intolerance [5]. Notwithstanding, a growing body of evidence has shown that autonomic imbalance characterizes a significant subset of CHF patients also in the case of preserved LVEF, contributing to disease progression and poor outcomes [4, 6, 7]. Interestingly, in this context, the interaction between the ANS and immune system (i.e., the neuroimmune cross-talk) seems to play a crucial role, contributing to the activation of pro-inflammatory, pro-oxidative, and pro-fibrotic cascades [8].

Therefore, autonomic imbalance and its detrimental consequences persist in many CHF patients despite therapeutic advances, fostering research for the development of further neuromodulation strategies [9]. Various approaches have targeted the SNS, mainly involving denervation, with inconclusive results [10]. Stimulating the PSNS represents a valuable alternative. Indeed, through either direct (i.e., on cardiac cells) or indirect (i.e., on pro-inflammatory pathways) effects, increasing cholinergic signaling may be beneficial to patients with CHF across the whole LVEF spectrum [11].

Nevertheless, the off-target effects of cholinergic drugs and the uncertain efficacy of bioelectronic devices have so far prevented their clinical translation [10, 12]. Transcutaneous vagus nerve stimulation (tVNS) is an emerging opportunity to achieve neuromodulation non-invasively but is still to be tested in large-scale trials [13, 14].

In this review, we aim to summarize the current knowledge about the role of the PSNS in CHF pathophysiology, describing the methods to evaluate vagal cardiovascular control and unraveling the rationale, limits, and perspectives of stimulating PSNS to improve outcomes.

## Cardiovascular parasympathetic control

Cardiovascular autonomic control relies on a series of reflex arcs composed of specialized peripheral receptors, efferent and afferent arms, and integrative centers [15].

Efferent axons of the ANS are organized into preganglionic and postganglionic fibers; for the SNS, the preganglionic axons are long and the postganglionic axons are short, whereas the reverse is true for the PSNS. Preganglionic neurons of the SNS are located within the lateral horn of the thoracolumbar spinal cord, whereas for the PSNS, these are located within the sacral spinal cord and in the brainstem. With respect to the heart, preganglionic neurons of the PSNS are located in the ventrolateral region of the nucleus ambiguous and in the dorsal motor nucleus and project within the vagus nerve [16]. These axons originate bilaterally within the caudal ventrolateral medulla and exit the brain via the jugular foramina [17].

The vagus nerve is a mixed nerve, composed of afferent (80–90%) and efferent (10–20%) fibers [18]. Though organized in different fascicles, at least in the cervical tract, the topography of vagal fibers in humans is still an object of study [18]. From the cervical and thoracic tracts of the vagus nerve arise the cardiac branches, which converge to the cardiac ganglia, being part of the intrinsic cardiac nervous system (ICNS) [18]. Within the ICNS, postganglionic neurons are organized in clusters, constituting functional circuits with sympathetic neurons and interneurons [19, 20]. Out of over 800 cardiac ganglia, seven subplexuses have been identified in humans: the dorsal and ventral right atrial plexuses, the dorsal and ventral left atrial plexuses, the middle dorsal plexus, and the right and left coronary plexuses [21]. Though functionally intertwined, atrial plexuses mainly modulate chronotropic and dromotropic functions, while coronary plexuses modulate ventricle contractility [21].

From the ICNS arise visceral afferent fibers, carrying mechanical, chemical, and nociceptive signals [22], whose somata are located in the nodose ganglion or the C6–T6 dorsal root ganglia [23]. The transduction properties of these fibers depend on their location but often display multimodal properties [24]. Furthermore, the activation of these fibers may result in either negative (i.e., inhibiting PSNS and activating SNS) or positive (i.e., activating PSNS and inhibiting SNS) responses [25].

Beyond cardiac fibers, the vagus nerve contains afferences from pulmonary arteries, aortic (left), and brachiocephalic artery (right) walls, carrying information from chemoreceptors and mechanoreceptors [26]. Though its physiological role is controversial, the auricular branch of the vagus nerve supplies sensory innervation to the acoustic meatus, the conchae, and the tragus. After traversing the temporal pyramid and engaging connections with the facial and glossopharyngeal nerves, these fibers reach the jugular ganglion [26].

Vagal afferents project to the nucleus tractus solitarius (NTS) and the area postrema of the medulla. The NTS has direct and indirect connections with cortical and subcortical structures (e.g., limbic areas, rostral ventrolateral medulla, intermediate lateral medulla, locus coeruleus) [18, 27] and is deemed responsible for modulating efferent pathways by integrating afferent signals [28].

### Role of acetylcholine on the heart

In cardiac ganglia, vagal fibers release acetylcholine (ACh), which binds to nicotinic receptors on postganglionic neurons, releasing ACh, which binds to muscarinic (M) receptors on cardiomyocytes. Growing evidence suggests that cardiac ganglia are integrative centers, in which parasympathetic and sympathetic signals modulate synaptic transmission, with contributions of interneurons and glial cells [29].

On cardiomyocytes, ACh induces negative chronotropic, dromotropic, inotropic, and bathmotropic effects [30], mediated by M2 and, less abundantly, M3 receptors [31]. The activation of M2 receptors results in (1) inhibition of adenylyl-cyclase, decreasing the activity of L-type Ca^2+^ channels, with negative dromotropic and inotropic effects [32], and “funny” current, with negative chronotropic effect [33, 34], and (2) stimulation of G_i_βγ subunit, activating the inwardly rectifying ACh-sensitive potassium channels (K_ACh_), with negative chronotropic, dromotropic, and bathmotropic effects [35]. Stimulation of M3 receptors, via a Gq protein, activates phospholipase C [36] reducing sinoatrial node firing [37]. Finally, M1 receptors modulate cardiac control with opposite effects to those of M2 receptors [38] and modulate norepinephrine release from sympathetic terminals [39].

Through all these actions, the PSNS exerts beneficial effects on cardiac function. Indeed, by reducing cytoplasmatic Ca^2+^ concentration, hyperpolarizing cardiac cells, and decreasing sympathetic activity, ACh reduces the risk of ventricular arrhythmias [40]. Furthermore, cholinergic stimulation contributes to the inhibition of pro-hypertrophic, pro-fibrotic, and pro-apoptotic cascades, by reducing the activation of the mitogen-activated protein kinase and transforming growth factor-β pathways and activating the phosphoinositide 3-kinase/Akt signaling [41, 42]. Beyond these direct effects of cardiomyocytes, ACh influences cardiac function also indirectly, by modulating the immune system, through the cholinergic anti-inflammatory pathway [43]. Although the precise mechanisms are unclear, vagal efferences seem to promote the homing of cholinergic T-cells in the spleen. These cells release ACh, which binds α7-nicotinic receptors on macrophages, favoring their shift to the anti-inflammatory phenotype and reducing the secretion of tumor-necrosis-factor-α and other inflammatory cytokines [44]. While the physiological impact of this reflex on cardiac function is unknown, its dysfunction, secondary to sympathovagal imbalance, may be crucial in disease conditions such as CHF [45]. Indeed, increased circulating levels of inflammatory cytokines and immune cell infiltrates have been reported in patients with CHF, in which they have been associated with clinical severity and risk of adverse events [46, 47].

Notably, all the effects of ACh are characterized by an instantaneous (“rapid-off”) modulation due to the presence of acetylcholinesterase (AChE) in the synaptic cleft, which is responsible for the dynamic transduction properties of PSNS activity [48].

## Methods of assessment of the parasympathetic nervous system

Muscle sympathetic nerve activity (MSNA) in accessible peripheral nerves is considered the gold standard for assessing sympathetic function [49]. However, given that cardiac sympathetic axons cannot be accessed by microelectrodes, measuring norepinephrine spillover into cardiac veins is the only means by which cardiac sympathetic drive can be assessed in humans [50]. Notably, there is a strong correlation between MSNA and noradrenaline spillover to the heart [51].

As for the PSNS, the responses to Ewing’s battery, heart rate variability (HRV), and baroreflex sensitivity (BRS) are used as indirect markers [52].

### Ewing’s battery

Developed to assess diabetes-related ANS dysfunction [53], the Ewing’s battery is also used in patients with CHF [54] and consists of evaluating heart rate and blood pressure responses to specific challenges (namely, Valsalva maneuver, standing-up, deep breathing, and sustained handgrip). According to the results of each test, a score is calculated to identify autonomic dysfunction [53].

### Heart rate variability

**Fig. 1:**
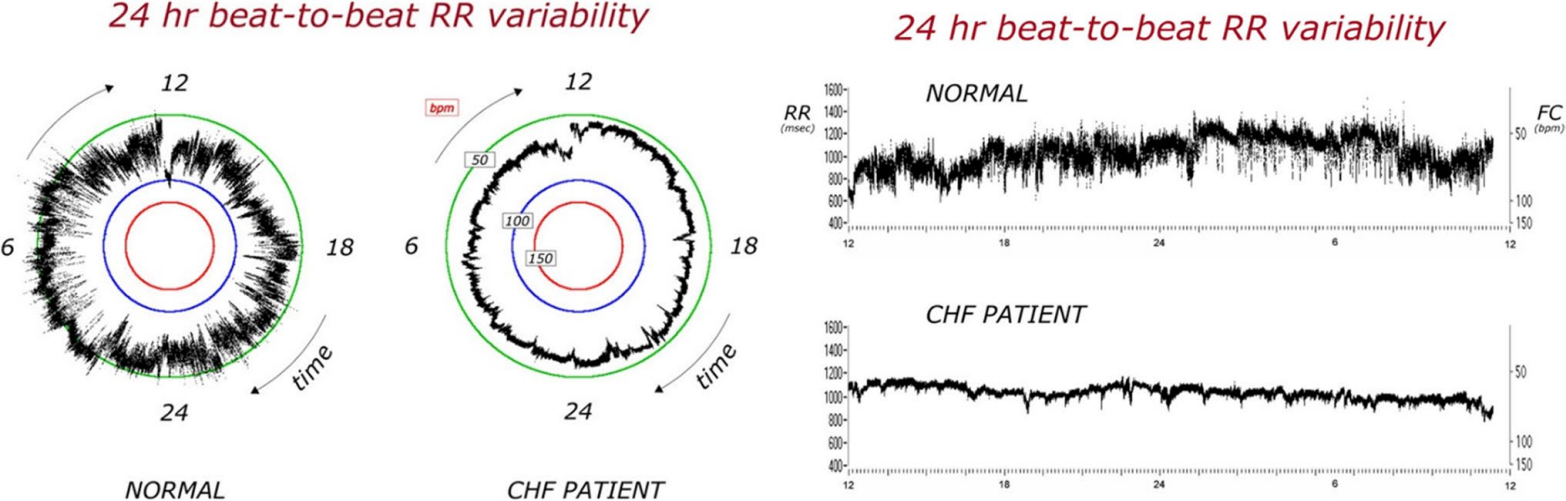
Heart rate variability in a healthy individual vs. a patient with chronic heart failure. As shown in the RR series, the patient with chronic heart failure (CHF) showed a significant reduction in either circadian or beat-to-beat heart rate variability (HRV)

HRV refers to the fluctuations in the time between consecutive heartbeats, reflecting the ability of the cardiovascular system to adapt to endogenous/exogenous changes (Fig. 1) [55]. A comprehensive description of HRV measures is provided elsewhere [55, 56]. Briefly, in resting conditions, heart rate shows beat-to-beat changes following linear patterns, due to ANS modulation, influenced by visceral feedback and triggered by changes in respiratory activity, vascular tone, body temperature, hormones, and circadian fluctuations.

HRV can be measured in its time and frequency domain [55, 57, 58]. Among the time-domain measures, the standard deviation of normal-to-normal (NN) intervals (SDNN) expresses the overall variability of heart rate. While the root mean square of successive NN interval differences (rMSSD) and the percentage of successive NN intervals that differ more than 50 ms (pNN50) reflect vagal modulation, no time-domain measure specifically expresses SNS modulation [55, 57–59].

The frequency-domain analysis relies on spectral methods to decompose the whole variability of a signal into frequency bands [60]. For heart rate, the main spectral components are high frequency (HF, 0.15–0.4 Hz), low frequency (LF, 0.04–0.15 Hz), and very low frequency (VLF, 0.003–0.04 Hz) [61]. While HF expresses vagal modulation mainly linked to respiratory sinus arrhythmia, both SNS and PSNS contribute to the LF component, centered on the 0.1-Hz oscillations, observable also at the vascular level (i.e., Meyer waves). Body temperature, hormones, and altered respiratory patterns (periodic breathing) contribute to VLF [61].

Several other HRV parameters have been proposed, including non-linear and entropy indices [56].

### Baroreflex testing

Arterial baroreflex modulates autonomic response to blood pressure changes. The reflex arc consists of stretch-sensitive receptors, mainly located in the carotid sinus in humans, an afferent arm to the brain via the glossopharyngeal nerve, and efferent vagal and sympathetic pathways [62]. Since the baroreflex-mediated modulation of sinus node activity mostly relies on rapid vagal signaling, BRS is considered a surrogate of PSNS function [15].

**Fig. 2:**
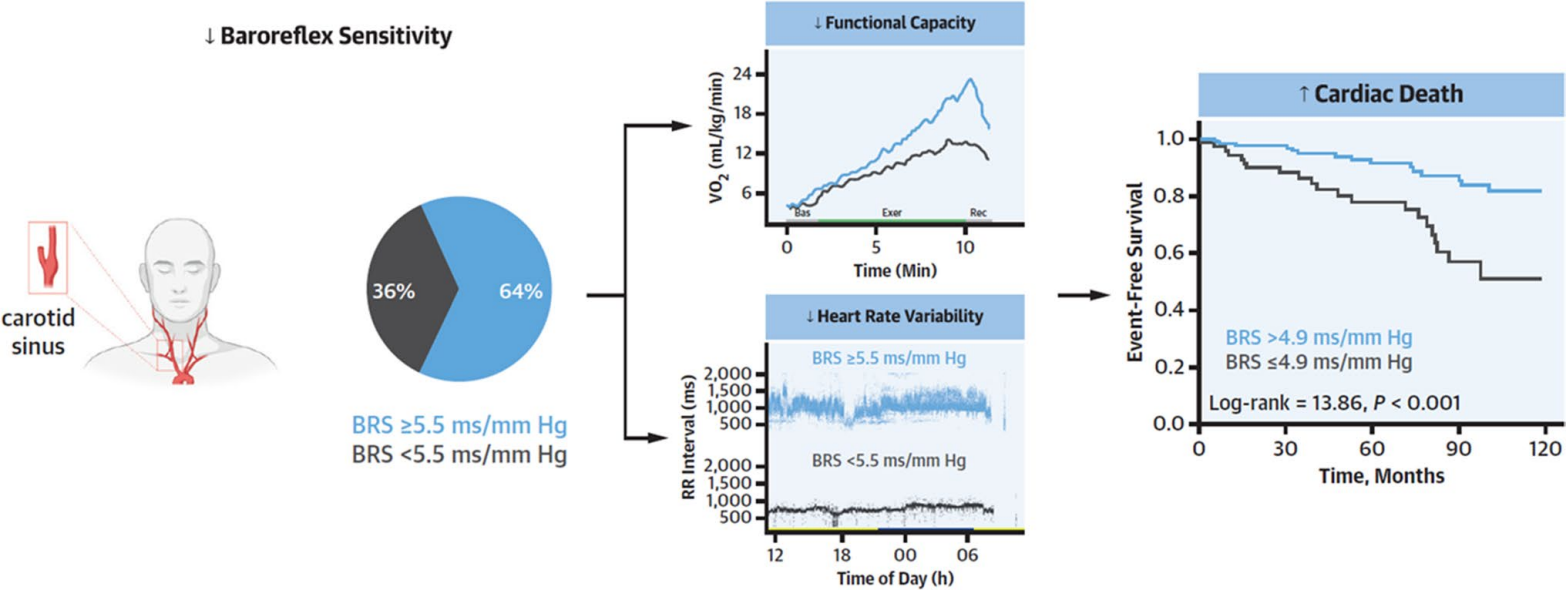
Clinical and prognostic significance of baroreflex sensitivity in patients with chronic heart failure. Abnormal baroreflex sensitivity (BRS) is frequently (36%) observed in patients with chronic heart failure (CHF) patients (*n* = 267), in which it is associated with functional impairment, lower heart rate variability, and a higher risk of cardiac death [9]

The use of pharmacological challenges to evoke controlled changes in blood pressure has been used to assess BRS [63, 64], showing prognostic value [65, 66]. To avoid the effects of drugs on end-organ function, different methods to assess the “spontaneous” BRS have been developed. The sequence method relies on the identification of the sequences of consecutive heartbeats with a parallel increase/decrease in heartbeats and blood pressure [67]. Other methods rely on the cross-correlations between the spectral components of heartbeats and blood pressure [67]. Though each of these measures showed predictive value, a simple ratio between the standard deviations of NN intervals and systolic blood pressure was the most accurate and reproducible among six methods [68] and a strong outcome predictor in a large cohort of CHF patients (Fig. 2).

Finally, a further possibility relies on the appliance of positive or negative pressure to the neck to assess the cardiac and vascular consequences of baroreceptors loading/unloading [69].

### Vagus nerve microneurography recording

**Fig. 3:**
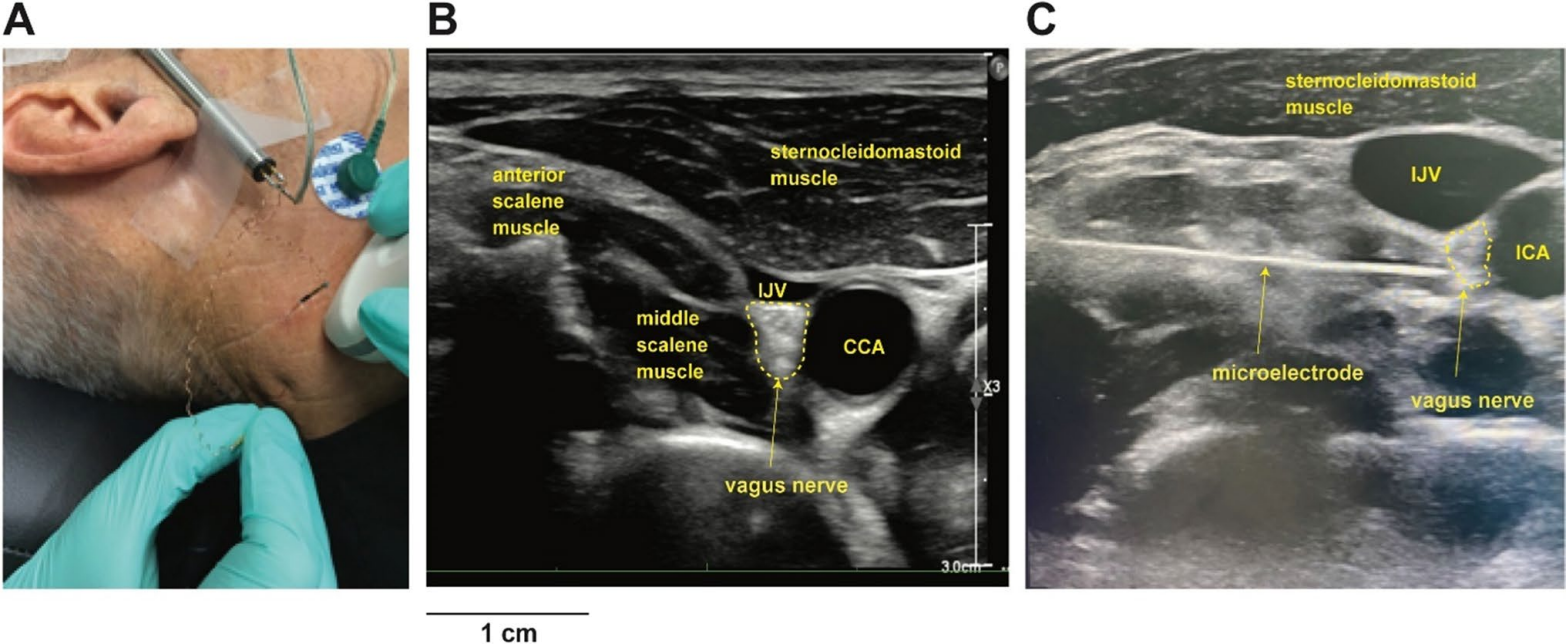
Microneurography recording of the vagus nerve. A tungsten microelectrode is inserted into the dorsolateral cervical region (**A**). Ultrasound guidance allows for the identification of the vagus nerve among muscular and vascular cervical structures (**B**). Direct visualization of the microelectrode through the probe allows for its precise direction toward the vagus nerve area, while avoiding vascular structures. CCA common carotid artery, ICA internal carotid artery, IJV internal jugular vein

While MSNA is the gold standard method to assess SNS function, only recently, the first recordings from the human vagus nerve were obtained through ultrasound-guided insertion of a tungsten microelectrode into fascicles of the vagus nerve in the neck (Fig. 3) [70]. This allowed the identification and classification of tonically active neurons directed to the sinoatrial node [70] and to document the cardiac and respiratory modulation of multiunit nerve activity [71]. Though preliminary, these findings pave the way for future experiments to study vagus nerve functions in health and disease. Indeed, given that it is possible to identify intrafascicular sites exhibiting cardiac rhythmicity, it will be very interesting to use this technique to identify changes in afferent and efferent vagal activity in CHF, for example.

## Parasympathetic dysfunction in heart failure

The neurohormonal model is a cornerstone of CHF pathophysiology. Accordingly, the pharmacological antagonism of SNS and RAAS has shown significant prognostic benefits and is a pillar of CHF treatment in patients with reduced LVEF [72].

Notably, sympathovagal imbalance is only partially reversed by current therapies. Indeed, increased MSNA and reduced HRV, sustained by feedback resetting, sleep disorders, and abnormal central control, feature in many CHF patients [73]. While increased MSNA showed a linear relation with disease severity and outcomes, the significance of abnormal vagal control is more controversial because of the absence of a gold standard marker [73]. Accordingly, most of the evidence derives from studies assessing HRV and/or BRS, which mirror the PSNS influence on the sinus node.

All the measures of HRV are depressed in CHF patients and related to disease severity and outcomes. Of note, patients with CHF and preserved LVEF show an intermediate phenotype between patients with reduced LVEF and healthy controls [74–76].

The underlying mechanisms are still to be completely understood. While the reduction in HF reflects blunted vagal modulation [77], also VLF [78] and LF components are often decreased or absent in CHF patients, proportionally to disease severity [77], and predict mortality [79]. Baroreflex desensitization and abnormal central control, also associated with cardiorespiratory interactions, are possible explanations [80, 81]. Decreased arterial compliance, alterations in sensory transduction, abnormal central mediation of the reflex, and efferent neurotransmission may be the mechanisms of reduced baroreflex function, while increased angiotensin-II and aldosterone signaling, as well as oxidative stress, are the proposed molecular pathways [82–86].

Beyond BRS, other sites of vagal dysfunction may be identified, from the generation of central outflow to ganglionic and postganglionic synapses to neurotransmission efficiency. In this respect, the presence of antibodies against the M2 receptors has been documented in patients with dilated cardiomyopathy [87] and associated with cardiac remodeling in rat models of CHF [88]. Nevertheless, a series of landmark studies showed that, despite the reduction of vagal tone and the response to electrical vagus nerve stimulation, the number and activity of cardiac M2 receptors are preserved or upregulated in CHF, while AChE is downregulated, probably as compensatory mechanisms [89–91]. On the other hand, an abnormal transmission at the ganglionic level was identified and proposed as a key mechanism [92].

Future studies, e.g., through direct vagal recordings, are expected to shed light on the complex mechanisms behind PSNS dysfunction in CHF patients.

## Targeting parasympathetic dysfunction in heart failure

**Fig. 4:**
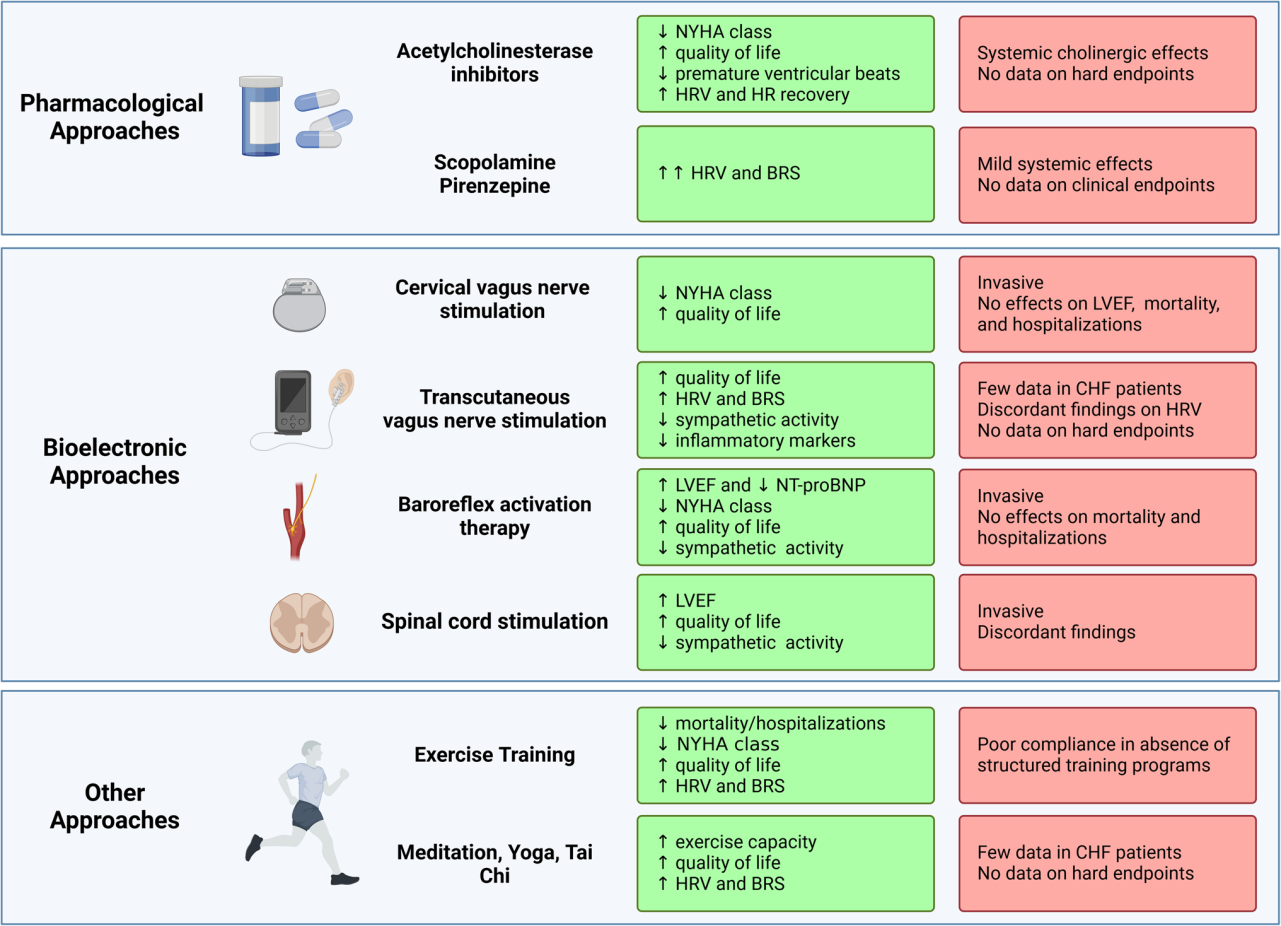
Vagal dysfunction as a therapeutic target in patients with chronic heart failure. Although different approaches have been already tested to improve vagal cardiovascular control in patients with chronic heart failure (CHF), no specific treatment has entered the clinical scenario so far. BRS baroreflex sensitivity, HRV heart rate variability, LVEF left ventricular ejection fraction, NT-proBNP N-terminal pro-B-type natriuretic peptide, NYHA New York Health Association. Created with BioRender.com

Targeting the PSNS represents an unmet need in CHF patients (Fig. 4). Indeed, beyond restoring neurohormonal balance, improving cholinergic stimulation also exerts anti-inflammatory actions, which is beneficial in CHF across the whole LVEF spectrum [11] and particularly in patients with preserved LVEF, in whom the efficacy of beta-blockers has been questioned [4, 8]. Nevertheless, the clinical trials conducted so far have not confirmed the expectations derived from preclinical and preliminary clinical studies (Tables 1, 2, and 3) [10].

**Table 1:** Clinical studies evaluating pharmacological approaches to target the parasympathetic nervous system in patients with chronic heart failure. *BRS* baroreflex sensitivity, *HF* high frequency, *HR* heart rate, *HRV* HR variability, *LF* low frequency, *NT-proBNP* N-terminal pro-B-type natriuretic peptide, *QOL* quality of life, *RCT* randomized controlled trial, *SDNN* standard deviation of normal-to-normal intervals

Drug/device	Study	Design	Population	Main findings
Pyridostigmine	Androne et al. 2003	RCT, vs. placebo	*n* = 20, LVEF ≤ 40%, NYHA I–III	↑ HR recovery after exercise
	Behling et al. 2003	RCT, vs. placebo	*n* = 23, LVEF < 45%, NYHA I–III	↓ ventricular ectopic beats; ↑ RR interval
	Serra et al. 2008	RCT, vs. placebo	*n* = 23, LVEF 29 ± 7%	↓ cholinesterase activity and chronotropic response; ↑ HR reserve, HR recovery after exercise, and oxygen pulse
	Villacorta et al. 2021	RCT, vs. ivabradine	*n* = 21, LVEF 33 ± 8%, NYHA I–III	↓ NYHA class, HR at rest, HR recovery, NT-proBNP, inflammatory markers ↑ QOL and oxygen consumption
Pirenzepine	Pedretti et al. 1995	RCT, vs. placebo	*n* = 20, LVEF 48 ± 6%, 19 ± 6 days post-MI	↑ all HRV measures and BRS
	Hayano et al. 1999	Single-arm, open-label	*n* = 30, LVEF 42 ± 9%, NYHA I–III	↑ RR interval, all HRV measures, and BRS
Scopolamine	La Rovere et al. 1994	Single-arm, open-label	*n* = 21, LVEF 23 ± 1%, NYHA II–III	↑ RR interval and HRV (SDNN and HF power); restoration of all the parameters after scopolamine withdrawal
	Venkatesh et al. 1996	RCT, vs. placebo	*n* = 12, LVEF 27 ± 9%, NYHA II–IV	↑ all HRV measures
	Casadei et al. 1996	RCT, vs. placebo	*n* = 23, LVEF 28 ± 2%, NYHA II–III	↓ HR at rest and submaximal exercise; ↑ HRV

**Table 2:** Clinical studies evaluating bioelectronic devices to target the parasympathetic nervous system in patients with chronic heart failure. *6MWD* 6-min walked distance, *ATN* aortic thoracic neuromodulation, *BAT* baroreflex activation therapy, *BRS* baroreflex sensitivity, *HF* high frequency, *HR* heart rate, *HRV* HR variability, *LF* low frequency, *LVEDD* left ventricular end-diastolic diameter, *LVEF* left ventricular ejection fraction, *NSVT* non-sustained ventricular tachycardia, *NT-proBNP* N-terminal pro-B-type natriuretic peptide, *NYHA* New York Health Association, *QOL* quality of life, *RCT* randomized controlled trial, *SCS* spinal cord stimulation, *SDNN* standard deviation of normal-to-normal intervals, *tVNS* transcutaneous vagus nerve stimulation, *VNS* vagus nerve stimulation

Drug/device	Study	Design	Population	Main findings
VNS	De Ferrari et al. 2011	Single-arm, open-label	*n* = 32, LVEF < 35%, NYHA II–IV	↑ 6MWD and LVEF; ↓ NYHA class
	ANTHEM-HF, 2014	RCT, open-label	*n* = 60, LVEF < 40%, NYHA II–III	↑ HRV, LVEF, and 6MWD; ↓ NYHA class
	NECTAR-HF, 2015	RCT, vs. stimulation off	*n* = 96, LVEF < 35%, NYHA II–III	↑ QOL; ↓ NYHA class; no effects on LVEF, remodeling, and NT- proBNP
	INOVATE-HF, 2016	RCT, open-label	*n* = 707, LVEF < 40%, NYHA III	↑ QOL and 6MWD; ↓ NYHA class; no effects on mortality or heart failure events
	ANTHEM-HFpEF, 2023	Single-arm, open-label	*n* = 52, LVEF > 40%, NYHA, II–III	↑ QOL and 6MWD; ↓ NYHA class and LF/HF; ↑ HR turbulence slope; ↓ T-wave alternans and heterogeneity; ↓ NSVT; no effects on cardiac function
	NCT03425422, stopped prematurely	RCT, open-label	*n* = 533, LVEF < 35%, NYHA II–III, LVEDD < 8.0 cm	Adverse events; LVEF, remodeling, NYHA class, 6MWT, QOL, HR, and HRV
tVNS	Tran et al. 2019	RCT, vs. sham tVNS	*n* = 24, LVEF > 49%	↑ frequency-domain HRV and ↓ global longitudinal strain
	Stavrakis et al. 2022	RCT vs. sham tVNS	*n* = 52, LVEF > 49% and 2 among obesity, diabetes, hypertension, ≥ 65 y.o	↑ QOL; ↓ global longitudinal strain and TNF-α; no effects on diastolic function; no effects on NYHA and BNP
	NCT02898181, ongoing	RCT vs. sham tVNS	*n* = 19, LVEF < 40%, in hospital phase	↓ IL-6 levels and endothelial cell oxidative stress. No effects on HR, blood pressure, and renal function
	NCT05789147, ongoing	RCT, open-label	*n* = 40, LVEF > 40%, NYHA II–III	HRV and BRS
	NCT05230732, ongoing	RCT, vs. sham tVNS	*n* = 158, LVEF < 40%	6MWD, QOL, HRV, C-reactive protein, NT-proBNP, TNF-α, and IL levels
BAT	Barostim HOPE4HF, 2015	RCT, open-label	*n* = 146, LVEF < 35%, NYHA III	↑ 6MWD and QOL; ↓NYHA class and NT-proBNP
	BeAT-HF, 2020	RCT, open-label	*n* = 408, LVEF < 35%, NYHA II–III	↑ 6MWD and QOL; ↓ NT-proBNP
	BeAT-HF, 2024	RCT, open-label	*n* = 332, LVEF < 35%, NYHA II–III	↑ 6MWD and QOL; ↓ NYHA class; no effects on mortality or heart failure events
	HF-FIM, 2022	Single-arm, open-label	*n* = 29, LVEF < 40%, NYHA II–III	↑ 6MWD, LVEF and QOL; ↓ NT-proBNP
ATN	NCT02633644, ongoing	Single-arm, open-label	*N* = 30, NYHA II–III	Adverse effects, NYHA, 6MWT, QOL, LVEF, remodeling
SCS	SCS-HEART, 2015	Single-arm, open-label	*n* = 21, LVEF 20–35%, NYHA III	↑ LVEF and QOL; ↓ NYHA class; ↔ NT-proBNP
	DEFEAT-HF, 2015	RCT, vs. stimulation off	*n* = 66, LVEF < 35%, NYHA III	No effects on remodeling QOL, 6MWD, NYHA class, all-cause mortality, or hospitalization for HF

**Table 3:** Clinical studies evaluating nonpharmacological/non-bioelectronic approaches to target the parasympathetic nervous system in patients with chronic heart failure. *BRS* baroreflex sensitivity, *HF* high frequency, *HR* heart rate, *HRV* HR variability, *LF* low frequency, *LVEF* left ventricular ejection fraction, *NYHA* New York Heart Association, *QOL* quality of life, *RCT* randomized controlled trial

Drug/device	Study	Design	Population	Main findings
Exercise	Coats et al. 1992	RCT, open-label	*n* = 17, LVEF 20 ± 2%, NYHA II–III	↑ HRV; ↓ norepinephrine spillover
	La Rovere et al. 2002	RCT, open-label	*n* = 95, LVEF 51 ± 13%, 28 ± 2 days post-MI	↑ BRS; ↓ 10-year cardiac mortality among responders
Meditation	Curiati et al. 2005	RCT, open-label	*n* = 19, CHF, LVEF 57 ± 14%, NYHA I–II	↓ norepinephrine and VE/VCO2 slope; ↑ QOL; no effects on cardiac function and oxygen consumption
Yoga	Krishna et al. 2014	RCT, open-label	*n* = 130, CHF, LVEF 30–50%, NYHA I–II	↓ HR, blood pressure, LF, and LF/HF ratio; ↑ HF

### Pharmacological approaches

Many of the drugs recommended for CHF treatment improve sympathovagal balance [93–96]. On the other hand, no molecule acting specifically on the cholinergic pathway is approved for this purpose (Table 1).

As reviewed elsewhere [97], AChE inhibitors showed protective effects in CHF rodents, by increasing HRV and BRS and reducing SNS activity [97]. Since some AChE inhibitors are used for neurological conditions (donepezil in the treatment of Alzheimer’s disease and pyridostigmine of myasthenia gravis), their cardiovascular effects have been investigated.

In two randomized, cross-over, double-blind studies, oral pyridostigmine improved heart rate recovery in 20 (30 mg, single dose) and 23 (45 mg, 3 t.i.d., for 1 day) CHF patients, respectively [98, 99]. In another study, pyridostigmine (30 mg, 3 t.i.d. for 2 days) lowered the incidence of premature ventricular beats and increased rMSSD and pNN50 in 20 CHF patients [100]. In a randomized, double-blind study, enrolling 21 CHF patients with heart rate > 70 bpm, pyridostigmine (30 mg, 3 t.i.d. for 6 months) reduced heart rate, natriuretic peptide levels, and inflammatory markers and improved symptoms and exercise capacity [101]. While the high rate of systemic effects (mainly gastrointestinal) due to systemic cholinergic stimulation may limit compliance to AChE inhibitors, the risk of long-term effects, including QT prolongation, is unknown in CHF patients [97]. While no studies have specifically investigated the effect of these molecules on clinical endpoints in patients with CHF, in an observational study on patients with Alzheimer’s disease without a history of CHF, the use of AChE inhibitors was associated with a significantly lower risk of new-onset CHF and cardiovascular death compared to propensity-score-matched non-users [102]. Similarly, in another observational study, patients with dementia treated with AChE inhibitors showed a lower risk of major adverse cardiovascular events, including heart failure-related hospitalization, compared with controls [103].

A potential alternative to AChE inhibitors is represented by two antimuscarinic agents, namely, scopolamine and pirenzepine, whose low-dose administration is associated with vagotonic effects, due to a greater affinity for M1 receptors, favoring ACh binding to M2 receptors and reducing norepinephrine release [104, 105]. The application of a scopolamine patch for 24 h in 21 CHF patients increased RR interval and HRV [106]. Similar findings were replicated in a randomized, cross-over, double-blind study, in which transdermal scopolamine increased HRV and BRS in 15 CHF patients [107]. Also, the intravenous administration of pirenzepine in 15 CHF patients increased SDNN and HF power [104]. The effects of these molecules were compared in a single-blind, placebo-controlled, cross-over trial in 20 post-myocardial infraction patients. While both drugs increased HRV and BRS, pirenzepine use yielded a lower rate of adverse effects (only 5% of patients reported nausea vs. 50% of patients reporting dry mouth, drowsiness, blurred vision, or nausea on scopolamine) [108].

Despite these findings, no study has investigated the efficacy of these molecules on clinical endpoints.

### Vagus nerve stimulation

Initially approved for drug-resistant epilepsy, VNS relies on an implantable neurostimulator activating cervical vagus fibers via a pulse-delivering electrode [109].

After the promising findings of preclinical research and preliminary human studies [110–112], VNS was tested in large-scale clinical trials, namely, the CardioFit [113], the ANTHEM-HF [114], the NECTAR-HF [115], the INOVATE-HF [116], and the ANTHEM-HFpEF study [117]. Despite some improvement in qualitative endpoints (e.g., symptoms and quality of life), no significant benefits were observed in terms of cardiac remodeling, neurohormonal activation, hospitalizations, and mortality [118]. Moreover, many patients experienced some discomfort secondary to VNS, while the risk of either procedural or long-term device-related complications raised safety concerns [118].

Although technological reasons (e.g., delivered currents, stimulation frequency, duty cycles) may have contributed to such results, the clinical efficacy of VNS has been questioned and the ANTHEM-HFrEF, designed to test a new-generation device, has been stopped prematurely for financial reasons (NCT03425422).

### Transcutaneous vagus nerve stimulation

A series of anatomical studies have shown that afferent fibers of the auricular branch of the vagus nerve may be found on the surface of the external ear [119]. The electrical stimulation of these fibers increased activity in central areas involved in autonomic control, including the ipsilateral NTS, dorsal raphe, locus coeruleus, contralateral parabrachial area, amygdala, and nucleus accumbens [119].

The cardiovascular consequences of tVNS have been studied almost exclusively in healthy individuals. While short-term tVNS increased HRV and BRS [120, 121], heterogeneous protocols and stimulation parameters contributed to the mixed findings reported by other works [122]. A single study showed that tVNS may reduce MSNA [123].

Based on the encouraging findings derived from preclinical studies of ischemic CHF models, in which tVNS improved cardiac remodeling and lowered arrhythmic risk [124, 125], tVNS was tested in patients with acute myocardial infarction, showing a reduction in ischemia–reperfusion injury [126]. Furthermore, in 50 patients with CHF and preserved LVEF, 1-h daily tVNS for 3 months improved left ventricular longitudinal strain and quality of life and reduced inflammatory markers [14], confirming and extending preclinical findings [127].

Further studies are ongoing to evaluate the effects of tVNS on various endpoints in CHF patients (Table 2).

### Baroreflex activation therapy

Considering the pathophysiological and prognostic significance of decreased BRS in CHF, enhancing this reflex has a solid rationale for improving outcomes [12]. Indeed, the direct activation of the afferent arm of baroreflex could maximize cardiovascular benefits, limiting off-target effects. Baroreflex activation therapy (BAT) involves a subcutaneous pulse generator with an extravascular lead placed on the carotid sinus. Developed to treat resistant hypertension, BAT increases markers of vagal control and reduces MSNA, with anti-remodeling cardiac effects [128, 129].

The clinical efficacy of BAT was tested in two randomized, open-label clinical trials, enrolling patients with CHF and reduced LVEF symptomatic despite therapies. In the Barostim HOPE4HF study (*n* = 146), BAT improved the distance walked, quality of life, symptoms, and neurohormonal activation [130]. Similar findings were obtained in the BeAT-HF (*n* = 408), leading to the approval of the device for clinical use [131]. Considering these findings, the use of BAT in selected patients was acknowledged in CHF guidelines [132]. While the safety and efficacy of BAT on symptoms and quality of life were confirmed in the postmarket phase of the BeAT-HF (*n* = 323, median follow-up 3.6 years/patient), the risk of cardiovascular mortality and hospitalizations did not differ between BAT and control [133].

An alternative to BAT is the implantation of an endovascular device into the carotid sinus to amplify the normal pulse-driven activation of carotid baroreceptors [134]. Developed for hypertensive patients, the Mobius HD device was tested in a small study (HF-FIM) enrolling 29 CHF patients, improving the distance walked, quality of life, LVEF, and natriuretic peptides [134].

A similar approach, but targeting aortic baroreceptors, is under investigation (NCT02633644).

### Spinal cord stimulation

The electrical stimulation of the dorsal spinal cord represents the most ancient bioelectronic neuromodulation strategy, first proposed for neuropathic pain and refractory angina [135]. Though the precise mechanisms remain unknown, this approach improved vagal tone and reduced SNS activity [135]. On the base of preclinical evidence [136, 137], spinal cord stimulation was tested in two trials enrolling patients with CHF and reduced LVEF. While it was effective on symptoms, quality of life, and cardiac remodeling in the SCS-HEART study (*n* = 22) [138], no significant benefits were observed in the larger (*n* = 66), randomized, and single-blind DEFEAT-HF study [139]. Differences in surgical approach and current delivery contributed to this discrepancy.

### Non-pharmacological and non-bioelectronic approaches

Exercise training represents one of the most efficacious ways to improve sympathovagal balance (Table 3). In 17 patients with advanced CHF, an 8-week exercise training improved HRV and norepinephrine spillover [140]. Furthermore, in patients with a history of myocardial infarction, a 4-week training period increased BRS, lowering cardiac mortality during follow-up [141]. The improvement in sympathovagal balance hence represents a key mechanism behind the benefits provided by physical training in CHF patients.

Yoga and meditation have beneficial effects on autonomic cardiovascular control, too. The slow breathing that characterizes these practices reduced heart rate and increased HRV and BRS in healthy subjects [142], while a downward modulation of chemoreflex sensitivity was described as well [143].

A few studies have evaluated these approaches in patients with CHF [144]. Among 19 CHF patients, meditation (two 1-h training sessions, followed by 30-min b.i.d. sessions for 12 weeks) reduced norepinephrine levels and improved quality of life and ventilatory efficiency, but did not affect oxygen consumption and LVEF [145]. Similar benefits were also attributed to yoga [146] and tai chi [147].

## Conclusions and perspectives

Despite therapeutic advances, the prognosis of CHF patients remains poor, with autonomic imbalance contributing to disease progression and life-threatening events. Implementing the current therapeutic armamentarium with novel neuromodulation strategies may prove valuable. Although enhancing vagal control holds promise, tailored pharmacological strategies remain underexplored, while technological uncertainties and conflicting findings have hampered the transition of bioelectronic devices into clinical scenarios.

Several issues should therefore be addressed [148]. A return to physiology seems mandatory to deepen our understanding of PSNS function in healthy and disease conditions. In this respect, direct recordings from the human vagus nerve offer a unique chance to optimize neuromodulation devices, shifting toward closed-loop protocols aligned with physiology to maximize benefits and minimize off-target effects. Battery duration, biocompatibility, and miniaturization are other obstacles to overcome.

In the meantime, abandoning the possibility of pharmacological PSNS stimulation, despite promising preliminary findings, appears a missed opportunity. Encouraging future studies to examine the efficacy of pirenzepine or scopolamine in CHF patients on modern treatments is hence warranted.

Finally, improving patient selection is paramount. Although neglected in the main trials conducted so far, assessing residual autonomic dysfunction, even by HRV and/or BRS, is crucial to identifying patients who could benefit from neuromodulation. Indeed, this is expected to maximize treatment effectiveness, limit biological and economic costs, and even decrease the number of patients needed for enrollment in clinical trials.

## Abbreviations

ACh: Acetylcholine
AChE: Acetylcholinesterase
ANS: Autonomic nervous system
BAT: Baroreflex activation therapy
BRS: Baroreflex sensitivity
CHF: Chronic heart failure
CNS: Central nervous system
HF: High frequency
HRV: Heart rate variability
ICNS: Intrinsic cardiac nervous system
LF: Low frequency
LVEF: Left ventricular ejection fraction
MSNA: Muscle sympathetic nerve activity
NTS: Nucleus tractus solitarius
PSNS: Parasympathetic nervous system
pNN50: Percentage difference between adjacent NN intervals > 50 ms
rMSSD: Root mean square of successive RR interval
SDNN: Standard deviation of NN intervals
SNS: Sympathetic nervous system
tVNS: Transcutaneous VNS
VLF: Very low frequency
VNS: Vagus nerve stimulation
